# Editorial

**Published:** 1863-11

**Authors:** 


### Editorial.

### MEDICAL COLLEGES IN THIS CITY.

The regular annual course of lectures in the Rush Medical College was commenced by a general introductory, delivered on the evening of the 1st of October, by E. Ingalls, M.D., Prof. of Materia Medica.

The lecture was well written, and contained many judicious suggestions to the class, and was listened to with pleasure by the audience. The number of students in attendance, was stated to be fully equal to that present at the commencement of any previous session.

The fifth annual course of instruction in the Chicago Medical College, began on the evening of the 12th of October. The general introductory lecture was delivered by the Professor of Principles and Practice of Medicine and of Clinical Medicine, and may be found in full in another part of this number of the EXAMINER. A larger number of matriculants were present than at the commencement of any previous term. After the lecture and the usual notices in relation to the several hours of lecturing, the hospital clinics, &c., the rooms of the new building were thrown open for examination.

All appeared to be well pleased with its arrangements, and with the general indications of a prosperous and useful collegiate year.

---

INDICATIONS OF PROGRESS.—The editors of the *Chicago Medical Journal*, neither of whom have paid the slightest attention to any medical society in this city, during the last ten years, nor taken any part in the State or National Associations for more than three years, are just now amusing their readers with editorials gravely setting forth the importance of medical societies and associated medical investigations. Is it possible that these gentlemen begin to realize the legitimate effects of their past indifference and selfish exclusiveness; and, especially, of their illiberal course towards the recent session of the American Medical Association in this city?—And do they hope quietly to escape from their present unenviable position under a profusion of editorial verbiage and pretended zeal for medical associations?

If so, we wish them speedy success; and hope they will immediately reduce their professions to practice, by either actively and cordially sustaining our present City, State, and National Medical Societies, or effecting the organization of others of a better character. We trust the various *Ship Canal* projects of the country are sufficiently matured to enable them to devote, at least, a part of their spare time to the interests of medical societies.

Chicago Medical Society.—This Society, which has maintained an active and useful career of more than ten years duration, holds its regular meetings in room No. 9, in Larmon's block, on Friday evening of each week. The meetings are well attended, and are devoted exclusively to the cultivation of medical science and practice. The reading of papers; the reports from the standing committee on the sanitary condition of the city; the discussion of questions specially selected for that purpose; and the relation of cases, occupy the time and constitute the doings of the Society.

At the meeting, on the evening of the 23d ult., Dr. Paoli presented an ovarion tumor recently extirpated from a patient, which was composed of several large cysts or sacs, from which was taken eight or ten gallons of serous fluid; and of several solid fibrous tumors aggregated together, weighing without the the serum, between six and seven pounds. The bulk of the solid part of the tumor rendered a long incision through the abdominal walls necessary, and though several adhesions existed, the extirpation was effected without much difficulty. The patient continued to progress favorably for the first few days, but subsequently, sunk under symptoms of peritoneal and phlebitic inflammation.

Dr. Davis, inquired whether any member of the Society had investigated the reported cases of ovariotomy, with a view to determine the relative fatality of cases in which the tumors were wholly made up of cysts containing fluid, as compared with those in which the tumors were partly solid and the fluid in the cystic portion thick and turbid? In the few cases, that had come under his own observation, those of the latter variety terminated fatally, while those of the former recovered. But those cases were too few to justify any practical inference, and he had made no general examination of the subject. He did not mean to convey the idea, that the difference in fatality depended on anything connected with the operation for extirpation of the tumors, but on the difference in the constitutional condition of the patients accompanying these two classes of cases. Remarks were made by several members, when a vote of thanks was tendered to Dr. Paoli, for the interesting pathological specimens presented; and the regular question for discussion taken up, as follows, "Is retarded disintegration or metamorphosis of tissue equivalent to positive nutrition?" The discussion related principally to the action of alchol on the healthy animal economy, and was continued with much interest until a late hour.

---

Matter for Reflection.—We copy the following letter from a recent number of the *Medical and Surgical Reporter*, of Philadelphia. The facts set forth need no comment from us. We commend them to the special consideration of the editors of the *American Medical Times*, and of the *Chicago Medical Journal*. When heartless tyranny takes the place of true efficiency; when official insolence is mistaken for professional dignity; and arrogant pretension supersedes scientific acquirements, then humanity is made to blush with shame:—

### UNNECESSARY AMPUTATION OF THE LEG—TETANUS—DEATH.

Washington, D.C., May 13, 1863.

J. V. P. Quackenbush, M.D., Surg.-Gen. S.N.Y.

Sir: Friday morning last, I was invited by a nephew of Senator Wilkinson, of Minnesota, to call at the National Hotel, in this city, to see Col. Newman, of the 31st New York Volunteers, who had reached there from the battle field, wounded. I called about nine o'clock a.m. No physician had been there; none had seen him since his arrival from the battle field. I found that he had been wounded in the left foot by a grapeshot, on Sunday, 3d of May. The ball had passed obliquely upward from the left side of the foot, crushing the anterior part of the tarsus and lodging just under the skin, but not involving the ankle joint. The ball had been removed, as the Colonel told me, from 12 to 15 hours after the injury was received. The surgeons, including the Division-Director, decided that the foot could be saved, and the Colonel was sent to this city on a stretcher, and arrived about an hour and a-half before I saw him.

The opening was about two and a-half inches transversely across the foot; the foot and leg nearly to the knee hot, dry, and shining with inflammation. No appearance of suppuration; painful. Notwithstanding this, I told the Colonel that I concurred entirely with the surgeon in front, as to the probability of saying the limb. I recommended quietude and cold applications; washed out the wound and dressed the foot. I met Senator Wilkinson soon after my return, and he called my attention to the case, expressed himself pleased that I had called, and hoped the foot of the noble Colonel might be saved.

In the evening of the same day I called again, but Colonel Newman informed me that an army surgeon had been in, and with an omnious shake of the head had said, that the foot must be amputated. I advised, as a friend, against amputation, and the Colonel was hopeful, very thankful for the encouragement, and desired to place himself in my charge. The foot and leg was yet in a high state of inflammation; the evaporation produced by the cold lotions had somewhat relieved the pain and tension and the inflammation was gradually subsiding. Each day until, and including the 11th, I called and washed and dressed the wound twice a-day. On the fourth day, the inflammation had very considerably abated, and suppuration had commenced. The wound in the skin and soft tissue had begun to granulate, the whole appeared healthy, and the constitutional symptoms had subsided. The Colonel's appetite was good, he slept well, and experienced little or no pain, except when the limb was moved. I had not changed my previously expressed opinion as to saving the limb, but the result of the treatment confirmed me in the belief that the chances of life were better without amputation than with. Dr. Spencer, of Watertown, Dr. Green, of New York city, and five army surgeons of good standing and experience, who saw the Colonel, and the wound, expressed opinions very similar to my own. The Colonel assured me that in several instances the same opinion that I had advanced was expressed, and that the chances of life were better by waiting than by amputating the limb.

On the 11th, I learned from the Surgeon-General of the United States, William A. Hammond, on whom I had called on business connected with my going to the army of the Potomac, that he objected to my visiting Col. Newman in any capacity, even as a friend,—that the National Hotel, at which the Colonel was stopping, was located in a certain district in Washington, and that an army surgeon had charge of the district, and that the patient belonged to such surgeon, and that I had no business to call in any capacity. The Colonel told me the same day, that he was fearful the army surgeons would take off his foot that day; they had told him the evening previous, that he must take a good night's rest, and he thought it was omnious of their intentions. I learned from another source that it had been determined to take off the Colonel's foot the following day, and I declined to call again. On the evening of the 11th, I received the following note:

Dr. Swinburne:—Dear Sir,—Will you oblige me by calling this evening. I learned, this afternoon, that some matters of professional etiquette would prevent your calling, and I therefore invite you. You were the first surgeon who visited me after my arrival in this city, and you gave me permission to call on you at any time, night or day. I take the liberty of holding you to your offer. Yours respectfully,

(Signed) Leopold C. Newman,
Lieut.-Col. 31st New York Volunteers.

National Hotel,

*Washington, D.C., May* 11, 1863.

In compliance with the note I called, with Dr. Spencer, of Watertown, N. Y., and found the Colonel in a state of great excitement. There had been a consultation of surgeons at his room that afternoon, and they had decided to operate, stating that they should have done so before had they not had so many cases to attend to. He was of the opinion that nothing short of taking off his foot would satisfy the surgeons, and they had assured him that he would be quite well in two or three weeks. The Colonel asked me what he should do. I advised him to get permission from the Department to continue his journey to New York, where he could have the counsel of his own physicians and surgeons. Dr. Spencer offered to accompany the Colonel, if he secured permission to go. Colonel Newman had succeeded in postponing the operation that day.

On the 12th, he requested permission to go home to New York, but the surgeons decided against it, and said that he must have his foot amputated or they would not attend to him, and that if he did not submit to their decision in regard to him, he would be reported to the Surgeon-General for contumely, and dismissed the service. The Colonel assured me that a friend of his had been so served. The evening of the 12th, when I called, the Colonel said he supposed he would be obliged to submit to the amputation to-morrow, the 13th, and that after the surgeons had accomplished their purpose, in a week or two, he would be permitted to go home. Surgeon McLean, of the 2d New York Volunteers, called with me on the evening of the 12th. I had been with him on his invitation to see Colonel

Parks, of the 2d New York Volunteers, whose right leg the surgeons had skilfully amputated some days previous. He was doing finely.

On the 13th of May, I did not call, but heard from Colonel Newman through a friend boarding at the National. Down to 12 o'clock m. the wound had not been dressed. The Colonel told my friend that he expected the surgeons would be there to amputate the foot that day; the Colonel had told me that he supposed there was no other way but to submit or be discharged the service in disgrace, without pay, as the surgeons had assured him he would be unless he did. A friend of the Colonel called on Dr. Clymer, who had been consulted in the case, and who appeared possessed with full power, and who gave his views after consultation with Surgeon-General Hammond on this case. Dr. Clymer told the Colonel's friend, and also my friend, that he would not give the Colonel the choice to go to New York or remain here; that he was only to remain here and have his foot amputated; that if he did not submit here they would leave him, and he should have neither pay nor medical attendance, but that they would strike him from the roll, and leave him outside; that he had the authority of the Surgeon-General U. S. A. for saying this; that he had no right to receive, nor had any outside surgeon the right to give medical advice in such a case. If he did receive it, they would strike him from the roll and turn him out, and that they would have nothing whatever to do with him. And he added to the Colonel's friend: "If I find a citizen surgeon in the room looking at any of my patient's, I'll kick him down stairs."

All of which, save the last sentence, corresponds with what the Colonel had stated to me on the evening of the 12th, in the presence of several gentlemen; he remarked, that all these things were threatened by Dr. Clymer, in his interview with him at the National Hotel.

The following note, received from a friend who watched the Colonel's case from day to day, will give a just surgical record of what occurred from the 13th to the time of his death:—

*Washington, D.C., June* 10, 1863.

My Dear Sir:—After you left, on the 13th ult., Col. Newman was troubled with but little pain, meanwhile his wound freely suppurated.

On the 16th, I think it was, the attending surgeon had ether administered to him, and what was called a "perfect examination" of his wound was thoroughly made. A small piece of leather and a bit of bone an inch in length, and a little less in width, near or from the joint of his foot, was wrenched off. It required an exertion of strength to take it out with the instrument. The next day he was attacked with *tetanus*, with intervening spasms; a blister and opiate were resorted to. Dr. Clymer said that his only chance of life lay in amputation, and that it *ought* to have been done when he (Dr. C.) originally proposed it, as in that case there would have been no danger.* Dr. Clymer performed the operation, assisted by surgeons De Witt, Swasey, Farrel, and Allen. The next day tetanus grew worse; day after, in spite of morphia and black drop, the spasms were dreadful. For two or three days he remained in this terrible condition, till an application of chloroform to the back of the neck along the side and sciatic nerve, gradually brought relief. Appetite good, color good, and perfectly conscious. He may literally be said, by aid of medicines, to have worn out the tetanus.

On Sunday morning, the 7th inst., he was attacked with secondary hemorrhage. A good nurse was present, and a surgeon was at the bar of the hotel, and although immediately arrested, the loss of a few ounces of blood turned the scale, and he died in three hours.

This noble soldier often expressed his thanks for your kindness, and could not convince himself that, in handling and dressing his wound, any hands were as soft and delicate in their touch as yours.

To bravery that knew no fear, he united the susceptibility and loving disposition of a child; and our hearts are nearly broken, and our spirits saddened at his departure. He sent for me as soon as the hemorrhage was known; but when I came he was too much exhausted to speak, and a pressure from his hand alone told that I was recognized. Comment is needless.

Respectfully, &c.

* Dr. Clymer and his associates stated to the Colonel that the reason why he did not amputate at first, was, that he did not have the time—and that, secondly, why he wished to amputate—the surgeons had not time to attend him through so long a period as he would require surgical attention, without amputation. Thirdly. If amputation was performed he could go home in three weeks, and be fitted with an artificial leg. On the 13th, when the operation was to have been performed, it was still further postponed to accommodate the surgeons; while on the 16th, the condition of the Colonel and his wounded limb was better than at any previous time after the primary stage, and was daily improving, and still Dr. Clymer says, "It (amputation) ought to have been done when he originally proposed it, as in that case there would have been no danger." Notwithstanding this assertion, the surgeons, instead of amputating on the 16th, administered ether and irritated the lacerated parts to such an extent as to produce "Tetanus."

POINTS OF INTEREST IN THIS CASE.

There are several points here worthy of note: 1st. The surgeons on the field decided upon the propriety of not amputating the foot of Colonel Newman; that it could be saved "without amputation." 2d. That the injury was inflicted on the 3d, and the surgeons on the field decided not to amputate. When he arrived in Washington, on the 8th, while the whole limb was tumefied and absolutely shining with inflammation, the surgeon in Washington wished to amputate. This was delayed from day to day, and still the foot improved in spite of the depression of mind caused by the constant threat of amputation. On the 13th, they demanded amputation, and it was delayed—the same condition of things exist, and I learn the surgeons decide upon waiting for a few days. On the 16th, Colonel Newman was troubled with little pain; meanwhile his wound freely suppurated, and in fact, his condition had continued to improve so that suppuration was free.

On the 16th, "The surgeons administered ether, and made a perfect examination" of what? Why, a wound that you could easily put all your fingers and thumb into.

This examination resulted (as the surgeons stated) in finding a small bit of leather, and in wrenching by great force a piece of crushed bone, about one inch square, from its connection with the living tissues, besides doing other irreparable injury to the soft parts. All of these loose bones, leather, &c., would have dropped out of the wound whenever loosened by nature. 3d. The 17th, (next day) he was attacked with tetanus—how significant!! Cause and effect are sure to follow. The story of the "apples" over again; you need not knock them from the tree, since if let alone they will fall when ripe; and so the bone will surely follow the organic laws of nature; *ergo*, the ignorant interference with the bone caused the irritation which resulted in tetanus; the amputation and hemorrhage followed; and the sequal—death—was the result. 4th. If amputation was to have been performed, why make the "examination" at all, in the manner it was made, since the eye could scan the entire wound, and the finger could easily pass through and into the wound and ascertain its condition? Then why irritate the parts before amputating at all? Since it is the desire of all good surgeons to avoid it, in order to save the shock, the pyæmia gangrene or tetanus. That the latter followed so soon after this injudicious interference, there need be no wonder.

Respectfully submitted,

JOHN SWINBURNE.

Chair of Medical Jurisprudence.—It should have been stated in the previous number of the Examiner, that H. G. Spafford, Esq., had resigned the chair of Medical Jurisprudence in the Chicago Medical College, which had been ably filled by him during the past four years. Inability to spare the necessary time from his professional practice was the cause of his retirement. The chair has been filled by the appointment of M. O. Heydock, M.D., of this city; whose professional attainments, general scholarship, and gentlemanly bearing, will render him not only a successful teacher but also a most agreeable member of the Faculty.

---

Ridgewood's Disinfecting Powder.—We have received the Report of a Committee of the New York Academy of Medicine, in relation to the value of this substance.

The Report was made by Dr. J. H. Griscom, and furnishes evidence that the committee, subjected the powder to a pretty thorough practical trial in various ways. The results of their investigations are clearly indicated in the following extracts from the Report:—

"The article called the "*Ridgewood Disinfecting Powder*," presented by a sample to the Academy of Medicine in July last, and referred to the Section on Public Health and Legal Medicine for examination and report, has been submitted to several experimental tests, which together with evidences of its value obtained from other sources, especially some of the U.S. military hospitals, will satisfy the Academy not only that it is a valuable addition to the class of substances denominated disinfectants and deodorizers, but that it also possesses decided antiseptic powers.

"The composition of the powder, as given by Mr. Napier, the Chemist and President of the company manufacturing it, is as follows:—

| | | |
|---|---|---|
| Carbolic acid, | 5 to 8 | per cent. |
| Sesquichloride of iron, | 2 | " |
| Lime, from magnesian limestone, | 5 | " |
| Silicate of alumina (in the form of Fuller's earth), | 75 to 80 | " |
| Prepared charcoal, or ground pumice stone, | 10 to 12 | " |
| Sulphate of potash or soda, | a trace. | |

"It will be observed that here is a mixture of six different substances, having no reaction upon each other, but possessing each some power of reaction upon some one or more of the products of putrefactive decomposition.

"The first named ingredient, carbolic acid, may be regarded as an impure creosote, and possesses the deodorizing and antiseptic properties of that substance, even, it is said, in a greater degree. It belongs to the class of chemicals known as hydrocarbons, a class varying in the proportion of their elemental constituents, and having affinities varying with these proportions.

"The second component of the Ridgewood powder, sesquichloride of iron, acts particularly as a deodorizer upon excrementitious matters, and others whose decomposition yields ammonia and its compounds, which are among the most abundant and offensive products in many instances. Like other compouds of chlorine, it *breaks up* ammoniacal gas; but unlike chloride of lime, it evolves no odor of its own. In this powder it is used also for the purpose of neutralizing the effect of the quicklime employed in taking up the carbolic acid. This lime would otherwise promote ammoniacal exhalations.

"When the powder is desired for strictly medicinal purposes, the lime and the salt of iron should be omitted from its composition.

"The Potter's clay or Fuller's earth, which forms the 'body' of the powder, is also a good deodorizer. It has the property in nature of retaining ammonia in the soil, to be given to the plant, as may be required. Its absorbent properties cause it to retain both moisture and gases, to which its deodorizing powers are no doubt due.

"The small quantity of charcoal is added as an aider in the absorbent process.

"The modicum of lime is present for the purpose of drying the Carbolic Acid, allowing the latter to assume a pulverulent form, without impairing its chemical properties. The Sulphate of Soda and Potash are merely adventitious ingredients. If they have an acid reaction, they are useful in case of the generation of large quantities of ammonia, but not particularly otherwise, and they are not depended upon to much extent.

"This new addition to our resources for avoiding unpleasant odors, preventing the evolution of deleterious gasses, and arresting decomposition, may therefore, by a slight variation in its constituent composition, be made applicable to different purposes, *i.e.* for medical use, it may have a maximum of carbolic acid, and a minimum of lime and sesquichloride of iron. For ordinary deodorizing and agricultural purposes, the acid should be decreased, and the other ingredients more largely apportioned."

"The principal requirements, therefore, in any substance, to be effective as a disinfectant, are:—

"1st. That it shall remove or obviate offensive effluvia.

"2d. That it shall prevent putrefactive fermentation, so that the offensive odor, being once removed, shall not recur from the same substance.

"3d. That it shall combine with and preserve, in fœcal and other matters, the elements which form the food of plants.

"4th. That it shall be of moderate cost, and easily procurable.

"5th. That it shall add nothing to the manure injurious or preventive of its action.

"Of these several indications, our own experiments have proved some to be well answered by the Ringwood Powder, and there is sound theoretical reason to believe the others to be equally so.

"Our investigations on this important subject naturally lead to considerations connected with the practical applications of disinfectants as the means of purification, and the prevention of diseases, in all civic and military localities. We forbear, however, to extend this report any further than to express the opinion, that the great sanitary advantage to be derived from the use of the Ridgewood, or other similar deodorizers, in latrines, and cesspools of cities, and during the removal of their contents, as well as in military hospitals, camps, barracks, &c., is at once apparent. But especially would it prove valuable in preventing the decomposition of dead bodies of men and animals on the field after a battle, or in cities which have been subject to long seiges and protracted military occupation, where vast accumulations of *debris* taint the air, and are the almost inevitable cause of endemic maladies of serious character."

---

CLINICAL LECTURE.—*Vaccination.*—Delivered at St. Mary's Hospital Medical School. By Graily Hewitt, M.D., Lecturer on Midwifery and Diseases of Women and Children. Continued.

*Is the protective power of vaccination affected by lapse of time?* This is a most interesting question, involving as it does the decision as to the necessity or otherwise of *revaccination*. It would appear that after the lapse of a certain number of years the protective power of vaccination has a tendency, more marked in some individuals than others, to wear out, and a resusceptibility to smallpox arises. The history of revaccination lends support to this view, which is, as Dr. Budd has recently very justly observed, supported by what physiology teaches in reference to the change and renovation of the body, it being the fact that about every seven years the body is physically completely changed and renewed.

Some statistics as to the history of revaccination in the Prussian Army (from Mr. Simon's work), must be mentioned in connection with this subject. For many years past it has been the custom to revaccinate every soldier admitted into the Prussian army. In 1833, the system of revaccination in the army was begun; and in that year the percentage of cases in which the vaccination took effect was 33. The percentage of revaccination success has progressively increased since that time, the percentages each year being represented by the following figures:—33, 39, 42, 46, 49, 50, 52, 54, 57, 58, 57, 58, 60, 64, 64, 64, 61, 64, 69, 69, 69, 70; so that the percentage has increased from 33 to 70 per cent.

These figures prove that in Prussia—a country in which, so far as is known, vaccination is well attended to—the first vaccination has been followed by a resusceptibility to the vaccine disease, which is not represented constantly by the same figure. These statistics lead probably to the view that the efficacy of the vaccine lymph used throughout Prussia has deteriorated in process of time. The latter is a point which will be specially referred to in the next lecture. But we are now concerned with another question: the possible wearing out of the protective power of vaccination against smallpox by lapse of time. It is reasonable to infer that if a resusceptibility to the vaccine disease be shown to arise in the individual, a like resusceptibility to smallpox will concurrently arise in that particular case; and the impression has been for several years past here gaining ground that revaccination is proper, and even necessary, once or more in the life of the individual. Mr. Marston states that during 17 years not a single servant or nurse belonging to the Smallpox Hospital has taken smallpox, and the universal custom has been to revaccinate the servants, nurses, and attendants in or about the hospital on entering on their employment at this institution. It does not appear that the goodness of the first vaccination necessarily extends the limit which time usually places on the vaccination as protective from susceptibility to the vaccine disease; in other words, an individual well vaccinated at first may be found as liable to take the vaccine disease after the lapse of 20 years as one imperfectly or badly vaccinated.

*For the time*, and probably in by far the majority of individuals, good vaccination is almost absolutely protective; but after the expiration of a time, which appears to vary in different individuals, which is influenced by idiosyncrasy or some unknown cause, a resusceptibility to the vaccine disease—and probably also consequently to the smallpox—arises, whether the primary vaccination have been good or bad. If the effect of good primary vaccination wear out in course of time, *a fortiori* must the effects of bad vaccination wear out proportionately sooner. The conclusion to be drawn as to revaccination is, that revaccination should be performed at the age of puberty, and perhaps again at the age of 25 or 30, and that it is desirable also to revaccinate at other ages than these during epidemics of smallpox, and especially in the case of individuals likely to come into contact with smallpox patients.

*History and nature of vaccination.*—For a full account of the early history of vaccination, and of the manner of which, step by step, our countryman Jenner arrived at a discovery which is emphatically *the* discovery of modery times, I must refer you to the numerous works on the subject, and especially to the Report on Vaccination, by Mr. Simon, before alluded to. Time will only allow me to remind you of the chief facts. Jenner performed his first vaccination in 1796, the subject of the operation being a boy aged 8. This child was afterwards inoculated with smallpox without effect. In 1798, Jenner published his essay on the subject. In 1801, 6000 individuals had been vaccinated; and Parliament voted Jenner the sum of £30,000 in recognition of the importance of his discovery.

The disease known as the vaccine disease, and which is witnessed on the udder of the cow, is in reality the smallpox affecting the animal. In this country, Mr. Ceely, of Aylesbury, and Mr. Bradcock, of Brighton, have proved that this is the case by inoculating cows with smallpox; and well-marked vaccinia has thus been produced. From cows so inoculated individuals have been vaccinated, the results being identical with those of vaccination with matter as ordinarily obtained from the cow. It is probable that if these facts had been known at the time Jenner made his discovery, much of the prejudice which he had to encounter would have been removed. Individuals had an objection to taking the disease of an animal, but there would naturally be less objection to taking a disease the identity of which with smallpox had been proved. Jenner surmised that it was so, as is evident from the name which he gave to the disease—"variolæ vaccinæ." The vaccine disease, then, is the smallpox which has passed through the cow, and which has, in so passing through the cow, become so modified that, when reconveyed to the human subject, it gives rise to a disease of exceedingly slight intensity, but which nevertheless is still the smallpox. For a full account of the vaccine disease I must refer you to the ever-to-be quoted paper in the "Transactions of the Provincial Medical and Surgical Association," Vol. VIII., by Mr. Ceely, of Aylesbury.

In the next place, we must consider *the phenomena produced by vaccination in the human subject.* The necessary outline of the subject now to be given must be completed by actual inspection and comparison of vaccinated cases, opportunity for which is afforded you at an adjacent public vaccination station. This practical study of the subject is absolutely essential.

*Vaccination for the first time.*—After the arm has been punctured, and the vaccine lymph introduced, the first effect is identical with that which would be observed if the lancet had been uncharged with lymph; it so remains during the next two days; and it is only on the third day, or towards the end of the third day, that appearances are observed about the wound of an unusual character. It then becomes red and slightly elevated. On the fifth day, the cuticle covering this little red spot is elevated into a small pearl-colored vesicle: and this vesicle evidently contains a fluid. The vesicle varies in shape, according to the shape of the incision made—it is round if a simple puncture be made; it is oval if there has been a small incision in the skin. On the eighth day—that is to say, at the close of the seventh day—the vesicle has become much larger; and it is then in perfection, as it is termed. The margin of the vesicle is turgid and elevated; the color is now somewhat yellow, not quite so transparent as it was, and, on close inspection, it is evident that the vesicle is cellular, and divided by septa, there being from 10 to 14 cells in each vesicle. Moreover, the vesicle is what is termed umbilicated, having a depression in the centre, over which part there is no elevation of the cuticle into bladders. After the eighth day, there is formed what is termed the areola, the skin around the vesicle becoming red, sometimes of a deep red tinge; the surface of this areola becomes tense, hot, and at the same time painful; the areola is usually circular. If the points of vaccination be close to each other, there will be a large red spot surrounding the whole, extending sometimes a considerable distance up and down the arm. An occasional but very rare result, and one which appears to be connected with the use of bad lymph, is inflammation of the cellular tissue of the arm.

The glands of the neck and armpit are usually slightly swollen. During the eighth and ninth, or even tenth day, the increase in the size of the vesicle goes on. It is extremely important that the two things—the *perfection* of the vesicle and its *greatest size* —should not be confounded. The vesicle is in perfection on the eighth day; but it is at its largest size on the ninth or tenth day. On the eleventh day, the vesicle begins to fade, the lymph within becomes absorbed, or the vesicle breaks, and it is discharged, and a scab forms, which becomes hard, dense, firm, and black, and finally falls off at a time which varies from the eighteenth to the twenty-first day after the performance of vaccination. There is left behind it a cicatrix, which is quite indelible if the vaccination be efficient. Such is the course of the disease normally.

Occasionally, there is observed what is termed "retardation" of the disease, and this may take place up to sixteen days, possibly even later. The arm does not "take" so early as it should do, although the disease, after it has begun to take, may go through the same course as that ordinarily observed. On the other hand, a too early taking of the vaccination indicates irregularity, and is usually due to some defective character of the lymph used.

*Secondary vaccination.*—As a rule, when vaccination has once been performed, the individual is no longer susceptible to the *regular* vaccinia. The effect of the vaccination is, under these circumstances, that irritation is set up analogous in *kind* to that observed in primary vaccination, but the disease sets in quickly, the redness often beginning immediately after the operation; the vesicles are imperfect; there is little evidence of presence of fluid; the duration of the effect is only five or six days (although variations in this respect may be noted), and the scar left behind is correspondingly small, and wanting in the typical characters. In quite exceptional cases the secondary vaccination takes perfectly, and the course of the second vaccination is more nearly identical with that of the first. These cases, in reality, resemble those in which has been observed the occurrence of smallpox after good vaccination.

*Characters of the cicatrix following good vaccination.*—It is important that you should be aware of the characters of a perfect scar, and note them accurately. The scar resulting from good vaccination is usually circular; it is radiated, indented, and foveated, having a number of little pits upon its surface; and it has a well-defined edge. Good cicatrices are generally of a considerable size. The size of a typical scar, such as would be observed after vaccination by the puncture of a lancet, which is the ordinary method, is that of a threepenny piece—just five-eighths of an inch in diameter.

Now, what are the characters of a defective scar? A defective scar is more or less wanting in all the characters mentioned. It is comparatively smooth; without indentation; without the little pittings; the edges are irregular and ill-defined, and it is often very small.

Lastly, with reference to the phenomena of vaccination, as ordinarily observed. Up to the seventh or eighth day, the child is perfectly well, but there generally set in, at this time, some constitutional disturbance and signs of irritative fever. This varies in degree in different cases; it is sometimes considerable.

It need hardly be observed that the wound inflicted by the vaccination is, like other wounds, liable to be affected by injurious conditions of the surrounding atmosphere. Pyæmia may follow the use of bad lymph; erysipelas has, in rare instances, been observed. Ordinary care is sufficient to prevent such evils. *Lancet, June* 13, 1863.

---

HYDROPHOBIA.—*Electricity.*—A patient suffering from hydrophobia, and so far advanced in the disease as to have become entirely unmanageable, was secured and bound to a mattress, and a copper-wire being wound around both feet, the conductor of the negative pole was attached to this wire; and the conductor of the positive pole, through a sponge saturated with vinegar and salt, was applied to the throat, and over the spine and body generally, with the full power of the battery. This caused instantaneous cessation of the spasms, and while under the influence of electricity the patient willingly drank liquids, and was free from the usual horror of them. The current was continued for some hours, with intervals of omission; the patient began to collapse, then vomiting and purging freely followed, and finally he fell asleep, and recovery ensued. This case, which occurred in New York, under the care of Dr. Lassing, seems extraordinary, and we should be glad to hear of a repetition of the experiment in England. (Dr. H. Lassing, page 50.)

---

NÆVUS.—*Tartar Emetic.*—Make a plaster of from 16 to 18 grains of tartar emetic, and 1 drachm of diachylon, and spread a considerable portion of this all over, and somewhat beyond the nævus, by means of the back of a strong knife, and keep it *in situ* by means of gummed paper. On the fifth or sixth day, the entire surface of the nævus begins to suppurate, a crust gradually forming, which passes off in about 15 days, leaving a small cicatrix. (Dr. Zeissl, page 132.)

---

Thapsus Verbascum.—Messrs. Bullock and Reynolds, of Hanover Street, sends us a specimen of the tincture of this plant, prepared as suggested in a former number of *The Lancet.* It is of a rich dark-brown color, almost as dark as laudanum, with a peculiar odor and taste, not disagreeable, and, we should think, well represents the properties of the plant.

On the same subject, Dr. Gardner writes to us:—"I wish you would remind the readers of *The Lancet* that the season is approaching for gathering the 'thapsus verbascum,' and preparing the tincture from the freshly-dried plant. I have found it so valuable a remedy for irritable coughs,—affording, in doses of half to one drachm, great relief, without the nauseating effects of ordinary expectorants, or the disagreeable narcotism of opium,—that I strongly recommend medical men who are troubled with cough to try it on themselves. I am sure many will be gratified with the result. It is best given alone, diluted with a small quantity of water."—*London Lancet.*

---

Cholera. — *Subcutaneous Injection of Morphia.* — Opium seems of undoubted benefit in cholera, but is so generally rejected immediately from either the stomach or rectum that its full effects cannot easily be obtained. If, however, it is injected beneath the skin, its action will be found to be prompt and efficient, relieving at once the violent abdominal cramps and muscular action of stomach and rectum. Gtts. xv. of liq. morp. acet. may be injected with a Wood's syringe, beneath the skin of the abdomen. (Dr. I. Ash, page 92.)

---

A New Method of Auto-Ophthalmoscopy.—M. Giraud Tuelon lately submitted to the Academy of Medicine of Paris, an instrument composed of two plain mirrors inclined one upon the other at angles of ninety-six degrees. The objective lens of the ophthalmoscope is placed before one of the mirrors, and before the other an ordinary ophthalmoscopic mirror. The left eye is then put in contact with the left mirror and the lens, the right eye with the ophthalmoscope or the mirror of the right side. A lamp is now placed on the right, as in ordinary exploration, and the auto-examination of the right eye is then very easy. M. Giraud Teulon has used the instrument upon himself with great success.—*London Lancet.*

